# Neonatal cytokines and chemokines and risk of Autism Spectrum Disorder: the Early Markers for Autism (EMA) study: a case-control study

**DOI:** 10.1186/1742-2094-11-113

**Published:** 2014-06-20

**Authors:** Ousseny Zerbo, Cathleen Yoshida, Judith K Grether, Judy Van de Water, Paul Ashwood, Gerald N Delorenze, Robin L Hansen, Marty Kharrazi, Lisa A Croen

**Affiliations:** 1 Division of Research, Kaiser Permanente Northern California, Oakland, CA 94612, USA; 2 (Retired) Environmental Health Investigations Branch, California Department of Public Health, Richmond, CA 94804, USA; 3 Division of Rheumatology, Allergy and Clinical Immunology, University of California at Davis, 451 Health Sciences Drive, Suite 6510, Davis, CA 95616, USA; 4 MIND Institute, 2825 50th Street, University of California at Davis, Sacramento, CA 95817, USA; 5 Department of Medical Microbiology and Immunology, 1 Shields Avenue, University of California at Davis, Davis, CA 95616, USA; 6 Department of Pediatrics, 2521 Stockton Boulevard, Suite 4100, Sacramento, CA 95817, USA

**Keywords:** Newborn, Cytokines, Chemokines, Autism spectrum disorders

## Abstract

**Background:**

Biologic markers of infection and inflammation have been associated with Autism Spectrum Disorders (ASD) but prior studies have largely relied on specimens taken after clinical diagnosis. Research on potential biologic markers early in neurodevelopment is required to evaluate possible causal pathways and screening profiles.

**Objective:**

To investigate levels of cytokines and chemokines in newborn blood specimens as possible early biologic markers for autism.

**Methods:**

We conducted a population-based case-control study nested within the cohort of infants born from July 2000 to September 2001 to women who participated in the prenatal screening program in Orange County, California, USA. The study population included children ascertained from the California Department of Developmental Services with Autism Spectrum Disorder (ASD, n = 84), or developmental delay but not ASD (DD, n = 49), and general population controls randomly sampled from the birth certificate files and frequency matched to ASD cases on sex, birth month and birth year (GP, n = 159). Cytokine and chemokine concentrations were measured in archived neonatal blood specimens collected for routine newborn screening.

**Results:**

Cytokines were not detected in the vast majority of newborn samples regardless of case or control status. However, the chemokine monocyte chemotactic protein-1 (MCP-1) was elevated and the chemokine Regulated upon Activation Normal T-Cell Expressed and Secreted (RANTES) was decreased in ASD cases compared to GP controls. The chemokines macrophage inflammatory protein-1alpha (MIP-1α) and RANTES were decreased in children with DD compared to GP controls.

**Conclusion:**

Measurement of immune system function in the first few days of life may aid in the early identification of abnormal neurodevelopment and shed light on the biologic mechanisms underlying normal neurodevelopment.

## Background

Recent epidemiologic studies estimate that 1 to 2% of children are affected by Autism Spectrum Disorder (ASD) [1-3]. Although autism was first described in 1943 [4], very little is known about the etiology of the vast majority of cases, and there are no definitive biological markers that clearly distinguish individuals with ASD from unaffected individuals.

Several epidemiological studies have reported immune dysregulation in children with ASD and their mothers, suggesting a role for the immune system in the pathology of the disorder [5-9]. Several research groups have found that individuals with ASD have increased neuroinflammation in brain tissues [10-12], imbalances in immunoglobulins, including increased levels of plasma IgG4 [13], reduced levels of total IgG [14] or reduced levels of IgG and IgM [15,16], and imbalances in cytokine/chemokine levels [17,18]. However, the mechanisms through which immune dysfunction may contribute to the etiology of autism are not understood [19].

Cytokines and chemokines are proteins involved in regulating hematopoiesis, inflammation, and immune cell proliferation and differentiation [20]. They also play an important role in normal neurodevelopment, including the processes of neuronal migration and synaptic plasticity [21,22]. These processes are tightly regulated and too much or too little of the signals mediated by cytokines can be detrimental to the developing fetus. For instance, in animal models, injection of the cytokine IL-6 or IL-2 into pregnant mice leads to neurodevelopmental abnormalities in the offspring including decreased prepulse inhibition and latent inhibition, attention, exploratory and social behaviors [23,24]. These findings suggest that reported associations between maternal infections or inflammation during pregnancy and ASD [25-31] could be mediated through a disruption in the balance of cytokine or chemokine levels.

The literature on cytokines/chemokines and ASD is expanding; however, results are sometimes inconsistent across studies [17,18,32-40]. The discrepancies are due to different research groups having utilized different types of bio-samples (serum, plasma, amniotic fluid), different age groups, different types of control groups (including siblings with likely inherited similarities with respect to ASD), non-standardized blood draws from differing seasons or times of day, and differences in length of time between biosample collection and ASD diagnosis (prenatal, newborn, post-ASD diagnosis). All of these factors may contribute to variability across studies in measured concentration of cytokines/chemokines and study findings [41] Among the reported results on cytokines and chemokines in relation to autism, very few studies have so far utilized newborn samples.

The objective of this new study was to examine the potential association between cytokine and chemokine profiles measured at birth and risk of Autism Spectrum Disorders and developmental delays.

## Methods

### Study population

The study population has been previously described [42]. Briefly, the sample was derived from the Early Markers for Autism (EMA) study, a population-based, nested case-control study designed to evaluate biologic markers of susceptibility and exposure in archived maternal mid-pregnancy and neonatal blood specimens from the same mother-baby pairs. The EMA population was drawn from the cohort of children born in California from July 2000 to September 2001 to women who were pregnant in Orange County, California, USA, and who participated in the State’s prenatal expanded alpha-fetoprotein screening program (XAFP). Three groups of children were identified: children with Autism Spectrum Disorder (ASD), children with developmental delay (DD) but not ASD, and general population controls (GP). Children with ASD or DD were ascertained from the California Department of Developmental Services (DDS), which operates a system of 21 Regional Centers (RC) that coordinate services for persons with ASD, mental disability, and other developmental disabilities. GP controls were randomly sampled from the birth certificate files after excluding all past or current DDS/RC clients and were frequency matched to ASD cases by sex, birth month and birth year at a 2:1 ratio.

### Diagnostic verification

After initial ascertainment of children with ASD or DD from records of the Regional Center of Orange County (RCOC), medical record abstractors compiled detailed diagnostic and clinical data from the RCOC records following a protocol initially developed by the Metropolitan Atlanta Developmental Disabilities Surveillance Program [43]. Expert clinical review of abstracted data was then conducted by a developmental pediatrician (RLH) to confirm the ASD or DD diagnoses for this study using the *Diagnostic and Statistical Manual of Mental Disorders, Fourth Edition* (DSM-IV) criteria. The final analytic sample consisted of 84 children with ASD, 49 children with DD but not ASD, and 159 GP controls.

### Specimen collection

Neonatal blood specimens were retrieved from the newborn screening specimen archives maintained by the California Department of Public Health. The neonatal specimen archive contains dried bloodspots collected for screening purposes on nearly every infant born in California (approximately 500,000 per year) since 1980. All newborn blood specimens for children included in this study were obtained by the heel-stick method, usually within 24 to 48 hours of birth. The blood specimens were collected at the nursery on a special S&S filter paper, and allowed to dry at room temperature prior to transport to the regional laboratory for routine screening for metabolic and other disorders. During transport, usually by courier from the hospital to a regional screening laboratory, the temperature of the specimens was not controlled. Blood spots remaining after routine testing were catalogued and stored at -20°C.

### Cytokine and chemokine measurements

Neonatal cytokine and chemokine concentrations were determined using a commercially available multiplex bead-based kit (BioSource Human Bead Kit; Invitrogen, Carlsbad, CA, USA). The following cytokines and chemokines were measured: IFN-γ, IL-2, IL-4, IL-5, IL-6, IL-1β, IL-8, IL-10, IL-12p40, TNF-α, granulocyte macrophage colony-stimulating factor (GM-CSF), IFN-γ-induced protein 10 (IP-10), monocyte chemotactic protein-1 (MCP-1), macrophage inflammatory protein-1alpha and 1beta (MIP-1α, MIP-1β), Regulated upon Activation Normal T-Cell Expressed and Secreted (RANTES), and C-C motif chemokine 11 (CCL 11 or eotaxin). The assays were carried out in accordance with the protocols provided by the manufacturer. Briefly, 50 μL of serum was incubated with anti-cytokine-conjugated beads in a 96-well filter-bottomed plate on a plate shaker. After two hours, the beads were washed using a vacuum manifold, and biotin-conjugated detection antibodies were added for one-hour incubation. Following a repeat of the washing step, beads were incubated with streptavidin phycoerythrin for 30 minutes. The plates were then read on a Bio-Plex 100 system (Bio-Rad Laboratories, Hercules, CA, USA) and analyzed using Bio-Plex Manager Software (Bio-Rad Laboratories, Hercules, CA, USA) with a five-point standard curve. Reference samples were run on each plate to determine assay consistency. All laboratory assays were conducted blinded to case-control status.

### Statistical analysis

Socio-demographic factors were compared between ASD, DD and GP groups using Chi-square test for categorical variables and *t*-test for continuous variables. For all values of analytes that were below the minimum detectable level (MDL) we assigned a value of MDL/2. Concentration of chemokines and cytokines were analyzed as untransformed, since natural log transformation of the values did not substantially change their distribution. All analyses were categorical and cut-points were based on the percent of study subjects with analyte values below the MDL. For analytes with < 25% sample below the MDL, we divided the observations into quartiles based on the distribution among the GP controls and used the lowest quartile as the reference. Values below the MDL were included in the lowest quartile. For analytes with 25 to 90% of the sample below the MDL, we created a binary variable and compared detected versus non-detected. Analytes with > 90% sample below the MDL were not considered for further analysis.

To investigate whether analyte concentrations at the extreme low or high end of the distribution predicted case status, we included two additional cut-points comparing values below the tenth percentile versus above the tenth percentile and values above the ninetieth percentile versus below the ninetieth, as was done in a previous study [32]. The 10th percentile cut-point was introduced when < 5% of GP samples were below the MDL. The 90th percentile cut-point was introduced when < 25% of the GP samples were below the MDL We conducted crude and adjusted analyses using logistic regression to estimate the risk of ASD and DD associated with each analyte separately. Covariates considered for adjusted models were gender (male, female), child birth month (calendar month), birth year (2000, 2001), maternal ethnicity (Hispanic, non-Hispanic), maternal place of birth (US, Mexico, other), maternal age at child birth (continuous in years), child age at blood draw (continuous in days), gestational age (<37 weeks, ≥ 37 weeks). Covariates were considered confounders and retained in the final model if individually they changed the odds ratio by 10% or more. Frequency matching variables (sex, birth month, and birth year) were included in all logistic regression models comparing ASD to GP control. Since the specimens were spread out on different plates, we additionally adjusted for laboratory plate as a categorical variable. Odds ratios were estimated for exposure categories with a minimum cell count of 5.

The study was approved by the institutional review boards of the California Health and Human Services Agency and Kaiser Permanente of Northern California.

## Results

There was no difference between ASD cases and GP controls in gestational age, age at newborn screening, or birth year. However, mothers of ASD cases were more likely to be non-Hispanic, born in the US, and to be slightly older compared to mothers of GP controls (Table 1). Children with DD were more likely than GP controls to be male, have their blood drawn slightly later and to be born in the year 2000 (Table 1).

**Table 1:** Demographic characteristic of Autism Spectrum Disorder (ASD) cases, developmental delay (DD) and general population (GP) controls - the Early Markers for Autism study

**Characteristics**	**ASD (n = 84)**	**GP (n = 159)**	**DD (n = 49)**	**Chi-square**	
	**N (%)**	**N (%)**	**N (%)**	***P*-values**	
				ASD versus GP	DD versus GP
Gender					
Male	73 (86.9)	139 (87.4)	29 (59.1)	0.89	**< 0.01**
Female	11 (13.1)	20 (12.6)	20 (40.8)		
Maternal ethnicity					
Hispanics	20 (23.8)	73 (45.9)	28 (57.1)		
Non-Hispanics	64 (76.2)	86 (54.1)	21 (42.9)	**< 0.01**	0.12
Maternal place of birth					
US	45 (53.6)	71 (44.7)	16 (32.7)	**< 0.01**	0.26
Mexico	9 (10.7)	58 (36.5)	22 (44.9)		
Other	30 (35.7)	30 (18.8)	11 (22.4)		
Maternal age (median and inter-quartile range)	31 (28 – 34)	28 (24 – 32)	29 (25 – 32)	**< 0.01**	0.68
Gestational age (GA in days)					
Preterm (GA < 260 days)	9 (10.7)	23 (14.5)	11 (22.5)	0.42	0.20
Term (GA ≥ 260 days)	75 (89.3)	136 (85.5)	38 (77.5)		
GA median and interquartile range	276 (269 – 280)	274 (267 – 279)	276 (265 – 282)	0.22	0.90
Child age (in days at newborn blood draw, median and inter- quartile range)	1.22 (1.02 – 1.76)	1.18 (1.03 – 1.56)	1.40 (1.05 – 2.27)	0.38	**0.03**
Birth year					
2000	23 (27.4)	31 (19.5)	26 (53)	0.15	**< 0.01**
2001	61 (72.6)	128 (80.5)	23 (47)		

For all cytokines and chemokines, the proportion of newborn specimens with analyte levels below the MDL was similar for children with ASD, children with DD, and GP controls (Table 2). IL-5, IL-8, IL-12p40, TNF-α, IP-10, MIP-1β and GM-CSF were not considered for further analysis because they were detected in < 5% of specimens.

### ASD versus GP controls

More than 50% of study subjects had levels of IFN-γ, IL-1β, IL-2, IL-4, IL-6 and IL-10 below the MDL (Table 2). ASD status was not associated with the detection of these analytes in newborn bloodspots (Table 3). There was no significant difference between ASD and GP groups in the levels of MCP-1 when comparing quartiles in crude and adjusted analysis. However, ASD cases were more likely than GP controls to have levels of MCP-1 above the 90th percentile (adjusted odds ratio (ORadj) = 3.24, 95% CI 1.41 to 7.47) (Table 3). We found no significant difference in levels of MIP-1α between ASD cases and GP controls when comparing quartiles or extreme values. For RANTES, the proportion of ASD cases with concentration in the fourth quartile was lower than that of GP controls (ORadj = 0.46, 95% CI 0.18 to 1.16). Moreover, a significantly higher proportion of ASD cases had concentrations of RANTES at or below the 10th percentile compared to GP controls (ORadj = 2.42, 95% CI 1.05 to 5.55) (Table 3).

**Table 2:** **Working range and proportion below the minimal detection level (MDL) for each analyte measured in newborn dried blood by case**-**control status - the Early Markers for Autism study**

**Analytes**	**Working range (pg/ml)**	**ASD Cases (n =84)**	**GP (n =159)**	**DD (n = 49)**
		**% below MDL**	**% below MDL**	**% below MDL**
MCP-1	13.03 – 9,160	19.0	18.0	26.3
MIP-1α	6.1 – 28,800	0.0	1.2	4.1
RANTES	22.36 – 19,100	0.0	0.6	2.0
Eotaxin	1.37 – 9,200	19.0	9.0	14.3
IFN-γ	0.58 – 11,370	77.4	73.0	86.0
IL-1β	2.5 – 5,470	77.4	76.8	79.6
IL-2	1.45 – 9,530	67.9	69.2	83.7
IL-4	2.21 – 14,470	77.4	74.2	86.0
IL-6	1.88 – 12,360	77.4	74.8	79.6
IL-10	3.4 – 22,350	53.6	54.1	51.0
IL-5	1.75 – 11,490	100	100	100
IL-8	6.23 – 13,630	97.6	98.7	98.0
IL-12p40	2.74 – 6,000	100	99.4	100
TNF-α	3.96 – 8,660	100	99.4	100
IP-10	2.01 – 1,420	100	100	100
MIP-1β	5.3 – 10,370	96.4	94.3	98.0
GM-CSF	0.88 – 5,760	98.8	99.4	97.9

ASD: Autism Spectrum Disorder; DD: developmental delay; GP: general population; MDL: minimal detection level.

**Table 3:** Crude and adjusted odds ratios with their 95% CIs Comparing levels of newborn blood spot cytokines/chemokines among children with Autism Spectrum Disorder (ASD), developmental delays (DD) and general population controls (GP) - the Early Markers for Autism study

**Exposure**	**Exposure categories**	**Developmental category**			**ASD versus GP**		**DD versus GP**	
		**ASD (N = 84) n (%)**	**DD (N = 49) n (%)**	**TD (N = 159) n (%)**	**Crude OR (95% CI)**	**Adjusted OR**^**a **^**( 95% CI)**	**Crude OR (95% CI)**	**Adjusted OR**^**b **^**(95% CI)**
MCP-1	Q1	27 (32.14)	17 (34.69)	41 (25.79)	Reference	Reference	Reference	Reference
	Q2	13 (15.48)	14 (28.57)	39 (24.53)	0.50 (0.23 – 1.12)	0.49 (0.20 – 1.20)	0.86 (0.37 – 1.99)	0.52 (0.16 – 1.71)
	Q3	15 (17.86)	9 (18.37)	39 (24.53)	0.58 (0.27 – 1.26)	0.48 (0.20 – 1.14)	0.55 (0.22 – 1.39)	0.45 (0.13 – 1.54)
	Q4	29 (34.52)	9 (18.37)	40 (25.16)	1.10 (0.55 – 2.17)	0.93 (0.43 – 2.01)	0.54 (0.21 – 1.35)	0.55 (0.15 – 1.97)
	≤ 90%	66 (78.57)	45 (91.84)	143 (89.94)	Reference	Reference	Reference	Reference
	> 90%	18 (21.43)	4 (8.16)	16 (10.06)	**2.43 (1.17** – **5.07)**	**3.24 (1.41** – **7.47)**	**-**	-
MIP-1α	Q1	23 (27.38)	22 (44.90)	41 (25.79)	Reference	Reference	Reference	Reference
	Q2	20 (23.81)	7 (14.29)	39 (24.53)	0.91 (0.43 – 1.92)	0.86 (0.36 – 2.02)	0.33 (0.12 – 0.87)	0.21 (0.05 – 0.78)
	Q3	19 (22.62)	13 (26.53)	42 (26.42)	0.80 (0.38 – 1.69)	1.01 (0.37 – 2.70)	0.57 (0.25 – 1.29)	0.36 (0.10 – 1.23)
	Q4	22 (26.19)	7 (14.29)	37 (23.27)	1.06 (0.50 – 2.20)	1.53 (0.52 – 4.49**)**	0.35 (0.13 – 0.92)	0.29 (0.06 – 1.35)
	>10%	70 (83.33)	14 (28.57)	143 (89.94)	Reference	Reference	Reference	**Reference**
	≤ 10%	14 (16.67)	35 (71.43)	16 (10.06)	1.78 (0.82 – 3.86)	1.72 (0.71 – 4.14)	**3.57 (1.59** – **8.01)**	**3.36 (1.16** – **9.69)**^**c**^
	≤ 90%	70 (83.33)	46 (93.88)	140 (88.05)	Reference	Reference	Reference	Reference
	> 90%	14 (16.67)	3 (6.12)	19 (11.95)	1.47 (0.69 – 3.11)	1.76 (0.70 – 4.39)	-	-
RANTES	Q1	26 (30.95)	21 (42.86)	40 (25.16)	Reference	Reference	Reference	Reference
	Q2	23 (27.38)	9 (18.37)	40 (25.16)	0.88 (0.43 – 1.80)	0.82 (0.37 – 1.81)	0.42 (0.17 – 1.04)	0.34 (0.10 – 1.15)
	Q3	20 (23.81)	14 (28.57)	40 (25.16)	0.76 (0.37 – 1.59)	0.63 (0.28 – 1.43)	0.66 (0.29 – 1.49)	0.55 (0.19 – 1.58)
	Q4	15 (17.86)	5 (10.20)	39 (24.53)	0.59 (0.27 – 1.28)	0.46 (0.18 – 1.16)	**0.24 (0.08** – **0.71)**	**0.14 (0.03** – **0.62)**
	> 10%	68 (80.95)	33 (67.35)	143 (89.94)	Reference	Reference	Reference	Reference
	≤ 10%	16 (19.05)	16 (32.65)	16 (10.06)	2.10 (0.99 – 4.45)	**2.42 (1.05** – **5.55)**	**4.33 (1.96** – **9.54)**	**3.78 (1.29** – **11.03)**
	≤ 90%	79 (94.05)	46 (93.88)	143 (89.94)	Reference	Reference	Reference	Reference
	> 90%	5 (5.95)	3 (6.12)	16 (10.06)	0.56 (0.20 – 1.60)	0.59 (0.18 – 1.89)	-	-
Eotaxin	Q1	25 (29.76)	17 (34.69)	41 (25.79)	Reference	Reference	Reference	Reference
	Q2	23 (27.38)	9 (18.37)	37 (23.27)	1.02 (0.49 – 2.09)	0.95 (0.42 – 2.14)	0.58 (0.23 – 1.47)	0.88 (0.27 – 2.85)
	Q3	19 (22.62)	12 (24.49)	42 (26.42)	0.74 (0.35 – 1.54)	0.58 (0.25 – 1.32)	0.68 (0.29 – 1.62)	0.62 (0.20 – 1.88)
	Q4	17 (20.24)	11 (22.45)	39 (24.53)	0.71 (0.33 – 1.52)	0.60 (0.20 – 1.79)	0.68 (0.28 –1.63)	1.11 (0.27 – 4.58)
	≤ 90%	76 (90.48)	44 (89.80)	143 (89.94)	Reference	Reference	Reference	Reference
	> 90%	8 (9.52)	5 (10.20)	16 (10.06)	0.94 (0.38 – 2.29)	1.10 (0.36 – 3.34)	1.01 (0.35 – 2.93)	1.24 ( 0.34 – 4.42)^c^
IFN-γ	Non-detected	65 (77.38)	42 (85.71)	116 (72.96)	Reference	Reference	Reference	Reference
	Detected	19 (22.62)	7 (14.29)	43 (27.04)	0.78 (0.42 – 1.46)	0.91 (0.47 – 1.75)	0.45 (0.18 – 1.07)	0.64 (0.23 – 1.76)^c^
IL-1β	Non-detected	65 (77.38)	39 (79.59)	122 (76.73)	Reference	Reference	Reference	Reference
	Detected	19 (22.62)	10 (20.41)	37 (23.27)	0.96 (0.51 – 1.80)	1.11(0.52 –2.35)	0.84 (0.38 – 1.85)	0.65 (0.19 – 2.23)
IL-2	Non-detected	57 (67.86)	41 (83.67)	110 (69.18)	Reference	Reference	Reference	Reference
	Detected	27 (32.14)	8 (16.33)	49 (30.82)	1.06 (0.60 – 1.87)	1.14 (0.38 – 3.43)	0.43 (0.19 – 1.00)	0.26 (0.01 – 8.76)
IL-4	Non-detected	65 (77.38)	42 (85.71)	118 (74.21)	Reference	Reference	Reference	Reference
	Detected	19 (22.62)	7 (14.29)	41 (25.79)	0.84 (0.45 – 1.56)	0.95 (0.49 – 1.85)	0.48 (0.20 – 1.15)	0.64 (0.23 – 1.80)^c^
IL-6	Non-detected	65 (77.38)	39 (79.59	119 (74.84)	Reference	Reference	Reference	
	Detected	19 (22.62)	10 (20.41)	40 (25.16)	0.87 (0.46 – 1.62)	1.01 (0.52 – 1.96)	0.76 (0.34 – 1.66)	0.91 (0.35 – 2.35)^c^
IL-10	Non-detected	45 (53.57)	25 (51.02)	86 (54.09)	Reference	Reference	Reference	Reference
	Detected	39 (46.43)	24 (48.98)	73 (45.91)	1.02 (0.60 – 1.73)	0.17 (0.02 – 1.55)	1.13 (0.59 – 2.14)	1.53 (0.68 – 3.48)^c^

^a^Results adjusted for maternal place of birth, child birth month, birth year, gender and specimen plate number.

^b^Results adjusted for maternal place of birth, child year of birth, gender, specimen plate number and child age at blood draw.

^c^Results adjusted for maternal place of birth, child year of birth, gender and child age at blood draw.

For MCP-1 and eotaxin, comparison between ≤ 10% versus > 10% was not possible because more than 5% of the sample was below MDL.

We found no significant association between levels of eotaxin and risk of ASD compared to GP controls in either crude or adjusted analyses (Table 3).

### DD versus GP controls

A higher proportion of children with DD had concentrations of MIP-1α and RANTES at or below the 10th percentile compared to GP controls (for MIP-1α, ORadj = 3.36, 95% CI 1.16 to 9.69; for RANTES, ORadj = 3.78, 95% CI 1.29 to 11.03) (Table 3).

We found no significant association between levels of MCP-1 or eotaxin and risk of DD compared to GP controls in either crude or adjusted analyses (Table 3).

## Discussion

Children with ASD were more likely to have increased levels of MCP-1 and decreased levels of RANTES in newborn bloodspots compared to GP controls. Children with DD had decreased levels of MIP-1α and RANTES compared to GP controls.

Our finding of increased levels of MCP-1 in ASD compared to GP controls contrasts with the study by Abdallah and colleagues [32], who reported no case-control differences in levels of MCP-1 measured in archived newborn blood samples from 359 ASD cases and 741 matched controls from Denmark. Our finding of no difference in concentration of MIP-1α between ASD cases and controls is similar to those previously reported by Abdallah *et al*. (2012a). Like our study, they also found reduced levels of RANTES in ASD cases compared to controls. However, in our study, this finding was not specific to ASD cases, as we found that levels of RANTES were also reduced in newborn blood specimens of children with DD compared to GP controls.

In the present study, the vast majority of cytokines were not detected in newborn blood specimens, and detection was not associated with case-control status. Our results are in contrast with those reported by Abdallah *et al*. (2012) [35] who found decreased levels of IFN-γ, IL-2, IL-4, and IL-6 and increased levels of IL-8 and soluble IL-6 alpha (sIL-6rα) in newborn dried blood spots from ASD cases and controls. Differences in laboratory techniques, study populations, and length of time between sample collection and sample processing may have led to the observed differences in results. In the present study, samples were collected from children born in 2000 and 2001 compared to the wide range in year of birth of study participants of previous studies.

Alterations in chemokine and cytokine levels in other specimen types have been previously reported. In an analysis of chemokine levels in amniotic fluid Abdallah *et al*. (2012) reported elevated levels of MCP-1 for children who were later diagnosed with autism compared to controls [34]. Whether the chemokines were of maternal or fetal origin is not clear. Furthermore, increased levels of MCP-1 in blood specimens collected from children after the date of ASD diagnosis was reported in a study of 2- to 5-year olds [17] that included 80 ASD cases and 37 typically developing controls.

MCP-1, RANTES and MIP-1α play a role in neuron development. While often associated with inflammation, sufficient levels of these chemokines are needed for healthy neuronal migration. Therefore, changes in levels during critical windows of development could alter neurodevelopmental outcome leading to either ASD or DD, as suggested by our data. Cytokines and chemokines have overlapping biology and functions [44]. They are known to be redundant and pleiotropic [45] which makes the determination of the exact roles of specific chemokines during neurodevelopment challenging [17]. Increased concentration of MCP-1 in ASD cases versus controls may be suggestive of an increased immunological active state in newborns who are subsequently diagnosed with ASD.

The results of this study should be considered in light of the following limitations. We were unable to validate the diagnostic status of autism by systematic clinical evaluation. Instead, we relied on expert review of information recorded as part of diagnostic eligibility for developmental services from regional center records. In addition, we did not have any clinical information such as infection status of children at birth or their mothers. Despite these limitations, the study was strengthened by the measurement of a panel of chemokines and cytokines in newborn blood samples, prior to when ASD or DD diagnoses were made, so it gives us a window into what was going on biologically during a critical period of neurodevelopment. The inclusion of the DD group allowed us to evaluate the specificity of the findings. We were also able to adjust our results by including several covariates in the multivariate analysis. Finally, our comparison group was matched to the ASD group.

## Conclusion

We found elevated levels of MCP-1 and decreased levels of RANTES in the newborn blood of children subsequently diagnosed with ASD. Levels of RANTES and MIP-1α were also decreased in children later diagnosed with DD compared to GP controls. If replicated in future studies, these findings suggest that measurement of immune system function in the first few days of life may aid in the early identification of particular neurodevelopmental trajectories. Further research on early biologic markers will be useful in understanding the mechanisms underlying early abnormal neurodevelopment.

## Abbreviations

ASD: Autism Spectrum Disorder; DD: developmental delay; DSM-IV: *Diagnostic Manual of Mental Disorders, Fourth Edition*; DDS: Department of Developmental Services; EMA: Early Markers for Autism; GA: gestational age; GM-CSF: granulocyte macrophage colony-stimulating factor; GP: general population; IFN: interferon; IL: interleukin; LOD: limit of detection; MDL: minimal detectable level; MCP: monocyte chemotactic protein; MIP: macrophage inflammatory protein; ORadj: adjusted odds ratio; RANTES: Regulated upon Activation Normal T-cell Expressed and Secreted; RC: regional center; RCOC: Regional Center of Orange County; sIL-6rα: soluble IL-6 alpha; TNF: tumor necrosis factor; XAFP: expanded alpha-fetoprotein screening program.

## Competing interests

The authors declare that they have no competing interests.

## Authors’ contributions

OZ contributed to the conception and design of the study, conducted statistical analyses, interpreted the data and was the primary writer of the manuscript. LAC contributed to the conception and design of the study, obtaining funding, acquiring data, interpreting the data, critical revision of the manuscript, and edited the manuscript. CKY managed the raw data. JKG contributed to the conception and design of the study, the interpretation of the data and critical revision of the manuscript. RLH contributed to the acquisition of data, the interpretation of the data and critical revision of the manuscript. PA contributed to the study design, the interpretation of data and critical revision of the manuscript. JVdW contributed to the design of the study, acquiring the data, the interpretation of data and edited the manuscript. GND contributed to the statistical analyses, interpretation of the data, and edited the manuscript. MK contributed to the conception and design of the study, obtaining funding, and acquiring data. All authors read and approved the final manuscript.
